# Supplementary material for: COVID-19 mortality rate and its associated factors during the first and second waves in Nigeria
Source: PLOS Glob Public Health. 2022 Jun 9;2(6):e0000169. doi: 10.1371/journal.pgph.0000169 (PMC10022313; doi:10.1371/journal.pgph.0000169)
Supplement: S1 Checklist — (DOC) [file pgph.0000169.s001.doc]

**STROBE Statement—Checklist of items that should be included in reports of *cohort studies***

|  | Item No | Recommendation |
| --- | --- | --- |
| **Title and abstract** | 1 | (*a*) Title available in lines 1-2; abstract available in lines 66-91. |
|  |  | (*b*) Abstract contains an informative and balanced summary of what was done and what was found. |
| Introduction | | |
| Background/rationale | 2 | The scientific background and rationale for the study are available in lines 93-128 |
| Objectives | 3 | Specific objectives in lines 128-131 |
| Methods | | |
| Study design | 4 | Study design is available in line 134-135 |
| Setting | 5 | A description of the study setting is available in lines 135-141; study dates including periods of recruitment are available in line 151. |
| Participants | 6 | The study eligibility criteria and methods of selection of participants are described in lines 152-169. |
| Variables | 7 | We defined the study outcome variable (COVID-19-related deaths) and survivor, in lines 170-177; however, all study covariates were defined in S1 Table. |
| Data sources/ measurement | 8* | The study data source is described in lines 142-151 as well as in Figure 1, which also demarcated the data sources for wave 1 and wave 2. |
| Bias | 9 | Our data management is an indication of efforts made to address potential selection bias; and the choice of missing indicator approach to handle missing data as outlined in lines 187-188 is another effort made to address bias. |
| Study size | 10 | Being an analysis of surveillance (secondary) dataset, we explained the reason for not formally estimating a sample size for the study in lines 150-151 |
| Quantitative variables | 11 | In lines 204-206, we explained the criteria used for the selection of variables for analyses. |
| Statistical methods | 12 | (*a*) In lines 190-213, we described all statistical methods utilised in this study, including the use of negative binomial regression used to control for confounding. |
|  |  | (*b*) Subgroups and interactions were not considered relevant for this study, so they were not examined. |
|  |  | (*c*) Missing indicator approach—lines 150-151 |
|  |  | (*d*) Regarding loss to follow-up, we excluded persons who died after 30 days of follow-up; also see line 176. |
|  |  | (*e*) Regarding sensitivity analyses, this study focused on the incidence of COVID-19 during the first and second waves in Nigeria. As such, all analyses were done by wave. |
| Results | | |
| Participants | 13* | (a) Figure 1 provides a comprehensive description of the numbers of individuals in each wave of the pandemic in Nigeria—e.g. numbers potentially eligible, examined for eligibility, confirmed eligible, included in the study, completing follow-up, and analysed. |
|  |  | (b) Figure 1 also provided reasons for non-participation at each stage of the selection process. |
|  |  | (c) Figure 1 is a flow diagram |
| Descriptive data | 14* | (a) Lines 222-230 and Table 1 describe the characteristics of study participants (eg demographic, clinical, social) in relation to the study outcome variable, and by waves. |
|  |  | (b) Throughout the study we presented information on the number of participants with missing data for each variable of interest. |
|  |  | (c) In lines 218-220, we provided a summary for the follow-up time in each wave. |
| Outcome data | 15* | Lines 223-224 report the numbers of outcome events during wave 1 and wave 2. |
| Main results | 16 | (*a*) Lines 264-293 describe unadjusted and adjusted estimates and their precision (e.g., 95% confidence interval). |
| Other analyses | 17 | S2 Table summarise the incidence rates of COVID-19 by Nigeria States |
| Discussion | | |
| Key results | 18 | In lines 299-302, we summarise key results with reference to study objectives. |
| Limitations | 19 | In lines 401-418, we discussed limitations of the study, taking into account sources of potential bias or imprecision, especially in relation to the surveillance quality in Nigeria. Where applicable, we also discussed both direction and magnitude of potential biases of our findings. |
| Interpretation | 20 | In lines 302-388, we provided a cautious overall interpretation of our results considering the predefined study objectives, limitations, and in relation to existing evidence. |
| Generalisability | 21 | We described the generalisability (external validity) of the study results in lines 398-407. We did this with transparency about the inherent limitation of the data source for the study. |
| Other information | | |
| Funding | 22 | There was no specific funding for this study. This has been noted on the online submission system. |

*Give information separately for exposed and unexposed groups.
