# Supplementary material for: COVID-19 mortality rate and its associated factors during the first and second waves in Nigeria
Source: PLOS Glob Public Health. 2022 Jun 9;2(6):e0000169. doi: 10.1371/journal.pgph.0000169 (PMC10022313; doi:10.1371/journal.pgph.0000169)
Supplement: S1 Table — (DOCX) [file pgph.0000169.s002.docx]

| **S1_Table 1: Incidence rates for COVID-19 death in the Nigerian States** | | | | | | |
| --- | --- | --- | --- | --- | --- | --- |
| **State** | **Wave 1 [N=65,524]** | | | **Wave 2 [N=90,390]** | | |
|  | **Death** | **Person-day at risk** | **Incidence rate**  **(95% CI) per 100,000** | **Death** | **Person-day at risk** | **Incidence rate**  **(95% CI) per 100,000** |
| **National** | 994 | 1,832,290 | 54∙25 (50∙98-57∙73) | 513 | 2,673,140 | 19∙19 (17∙60-20∙93) |
| **South-west**  Ekiti  Lagos  Ogun  Ondo  Osun  Oyo | 5  138  29  36  18  36 | 9,590  673,740  57,120  46,370  26,200  100,340 | 52∙12 (21∙70-125∙23)  20∙48 (17∙34-24∙20)  50∙77 (35∙28-73∙05)  **77∙63 (56∙00-107∙63)**  68∙70 (43∙28-109∙04)  35∙88 (25∙88-49∙74) | 5  1  16  22  4  76 | 15,870  897,830  71,920  43,470  43,550  90,220 | 31∙50 (13∙11-75∙69)  0∙11 (0∙02-0∙79)  22∙25 (13∙63-36∙31)  50∙61 (33∙32-76∙86)  9∙19 (3∙45-24∙47)  **84∙24 (67∙28-105∙48)** |
| **South-south**  Akwa-Ibom  Bayelsa  Cross-River  Delta  Edo  Rivers | 7  21  6  50  108  61 | 8,650  11,760  3,670  34,480  77,040  78,450 | 80∙92 (38∙58-169∙74)  **178∙56 (116∙43-273∙87)**  163∙71 (73∙55-364∙40)  145∙00 (109∙90-191∙31)  140∙18 (116∙09-169∙27)  77∙75 (60∙50-99∙93) | 1  5  3  21  51  21 | 35,580  12,510  6,760  22,230  54,800  116,750 | 2∙81 (0∙40-19∙95)  39∙97 (16∙64-96∙02)  44∙36 (14∙31-137∙53)  **94∙46 (61∙59-144∙88)**  93∙06 (70∙72-122∙45)  17∙99 (11∙73-27∙59) |
| **South-East**  Abia  Anambra  Ebonyi  Enugu  Imo | 7  16  37  22  11 | 27,550  7,600  32,710  38,620  16,960 | 25∙41 (12∙11-53∙30)  **210∙64 (129∙04-343∙82)**  113∙13 (81∙97-156∙14)  56∙97 (37∙51-86∙52)  64∙87 (35∙92-117∙13) | 7  0  4  4  22 | 12,350  46,630  19,830  28,120  28,710 | 52∙42 (24∙99-109∙95)  -  20∙17 (7∙57-53∙74)  14∙23 (5∙34-37∙90)  **76∙63 (50∙46-116∙38)** |
| **North-central**  Benue  FCT  Kogi  Kwara  Nasarawa  Niger  Plateau | 8  79  0  25  11  10  28 | 14,850  171,170  90  29,860  13,890  7,700  106,660 | 53∙86 (26∙94-107∙70)  46∙15 (37∙02-57∙54)  -  83∙73 (56∙58-123∙92)  79∙19 (43∙85-142∙99)  **129∙90 (69∙90-241∙43)**  26∙25 (18∙13-38∙02) | 11  69  -  27  22  5  6 | 23,390  393,660  -  60,370  50,990  19,350  157,910 | **47∙03 (26∙05-84∙93)**  17∙53 (13∙84-22∙19)  -  44∙72 (30∙67-65∙21)  43∙14 (28∙41-65∙52)  25∙84 (10∙76-62∙09)  3∙80 (1∙71-8∙46) |
| **North-west**  Jigawa  Kaduna  Kano  Katstina  Kebbi  Sokoto  Zamfara | 8  39  48  20  5  17  1 | 9,500  77,380  45,480  27,410  2,580  4,070  2,320 | 84∙19 (42∙10-168∙35)  50∙40 (36∙82-68∙98)  105∙55 (79∙54-140∙06)  72∙95 (47∙07-113∙08)  194∙06 (80∙77-466∙24)  **417∙33 (259∙44-671∙32)**  43∙09 (6∙07-305∙93) | 4  9  52  7  6  1  1 | 5,480  175,590  62,350  32,980  8,730  19,040  4,070 | 73∙05 (27∙42-194∙62)  5∙13 (2∙67-9∙85)  **83∙39 (63∙55-109∙44)**  21∙22 (10∙12-44∙52)  68∙70 (30∙87-152∙93)  5∙25 (0∙74-37∙29)  24∙55 (3∙46-174∙32) |
| **North-east**  Adamawa  Bauchi  Borno  Gombe  Taraba  Yobe | 19  12  27  21  1  7 | 6,040  10,590  22,390  26,610  1,130  1,700 | 314 (200∙63-493∙13)  113∙28 (64∙33-199∙47)  120∙58 (82∙69-175∙82)  78∙91 51∙45-121∙03)  88∙14 (12∙42-625∙74)  **412∙37 (196∙59-864∙99)** | 9  3  0  14  3  1 | 23,190  20,400  16,280  30,090  15,250  5,890 | 38∙81 (20∙19-74∙59)  14∙71 (4∙74-45∙60)  -  **46∙53 (27∙55-78∙56)**  19∙68 (6∙35-61∙01)  16∙99 (2∙39-120∙62) |
| Highest incidence rates are in **bold** | | | | | | |
