# Supplementary material for: COVID-19 mortality rate and its associated factors during the first and second waves in Nigeria
Source: PLOS Glob Public Health. 2022 Jun 9;2(6):e0000169. doi: 10.1371/journal.pgph.0000169 (PMC10022313; doi:10.1371/journal.pgph.0000169)
Supplement: Attachment — Submitted filename: LETTER TO THE EDITOR ON FIGURE LICENCE.docx [file pgph.0000169.s003.docx]

**Dear Editor-in-Chief,**

PLOS Global Public Health

**Response to the appropriateness of the licence for our figure shapefiles**

Thank you for reaching out to us on the appropriateness of the licence for our figure3 and 4. We apologise for the confusion with our previous response. We can confirm that the source of the shapefiles used to generate the maps shown in Figure 3 and 4 are in fact locally sourced from GRID3 Nigeria (<https://grid3.gov.ng>) (which can also be found from parent website GRID3 (<https://data.grid3.org>). Previously, we mentioned it was from the GADM – we apologies for this error (the shapefiles i.e., States and Local Authorities were not from there but from GRID3 Nigeria). The authors confirm that the maps from GRID3 Nigeria/GRID3 are under the CC BY 4.0 license, which allows unrestricted use and distribution, even commercially, by third parties.

Yours sincerely,

Kelly Elimian, for the co-authors
