# Supplementary material for: COVID-19 mortality rate and its associated factors during the first and second waves in Nigeria
Source: PLOS Glob Public Health. 2022 Jun 9;2(6):e0000169. doi: 10.1371/journal.pgph.0000169 (PMC10022313; doi:10.1371/journal.pgph.0000169)
Supplement: Attachment — Submitted filename: RESPONSES TO EDITORS COMMENTS.docx [file pgph.0000169.s004.docx]

**RESPONSES TO EDITORS’ COMMENTS**

**General remarks**

*We are extremely grateful to the Editor’s handling of the manuscript and the opportunity to address some comments raised in a revised manuscript. The Editor’s comments are in bold while our responses are in italics.*

**RESPONSES TO INDIVIDUAL COMMENTS FROM THE EDITOR**

**Corrections in the abstract: I appreciate the efforts of the authors to address all the reviewer's comments. However, it is necessary that all corrections made in the manuscript are adapted for the abstract. For example, the study type remains incorrect in the abstract. In addition, the abstract does not include all the analyses performed and the main study results.**

*R: We are very grateful to the Editor for highlighting the appropriateness of the study design in the initial comments and the need to reflect the changes made in the abstract. We have now made this correction in the methods section of the abstract, as indicated in the track changes (lines 70-72). Regarding the analyses performed, we had aimed to minimise word counts by omitting specific analysis; however, following your comments, we have now included in the methods section of the abstract the definition of COVID-19 mortality incidence rates (lines 74-77), as well as the plotting of Kaplan-Meier curves and geospatial analysis (lines 77-79). Thanks to the Editor’s comments, we believe that the revised abstract of the manuscript is more informative for readers to gauge the research methods and conclusions. We have read the results section of the abstract and think the key findings have been presented, in line with the study objectives and methods.*

**Journal Requirements**
*R: Thank you for updating us on the co-authors that had not confirmed authorship of the manuscript. I can attest to the fact that some of them did complete the confirmation process, but it appears the submission was not complete. Like the last time, I followed up with them to get authorship confirmed; however, I could not reach XXX despite sending an email (previously and yesterday) and making phone calls. To adhere to the journal’s guidelines for authorship, we have decided to drop XXX from the authorship list.*

**Reference list: Please review your reference list to ensure that it is complete and correct. If you have cited papers that have been retracted, please include the rationale for doing so in the manuscript text, or remove these references and replace them with relevant current references. Any changes to the reference list should be mentioned in the rebuttal letter that accompanies your revised manuscript. If you need to cite a retracted article, indicate the article’s retracted status in the References list and also include a citation and full reference for the retraction notice.**

*R: Thank you for highlighting the need to update our reference list. We have checked the list again and can confirm that we did not cite papers that have been retracted; and all the cited references remain relevant to the manuscript’s contents.*

**Figure formatting: While revising your submission, please upload your figure files to the Preflight Analysis and Conversion Engine (PACE) digital diagnostic tool,** [**https://pacev2.apexcovantage.com/**](https://pacev2.apexcovantage.com/)**. PACE helps ensure that figures meet PLOS requirements. To use PACE, you must first register as a user. Registration is free. Then, login and navigate to the UPLOAD tab, where you will find detailed instructions on how to use the tool. If you encounter any issues or have any questions when using PACE, please email PLOS at** [**figures@plos.org**](mailto:figures@plos.org)**. Please note that Supporting Information files do not need this step.**

*R: Our figures have been processed via the PACE platform, in line with the journal’s guidelines.*
